# Bioactive peptide inhibits acute myeloid leukemia cell proliferation by downregulating ALKBH5-mediated m^6^A demethylation of EIF4EBP1 and MLST8 mRNA

**DOI:** 10.1007/s13402-022-00666-9

**Published:** 2022-05-17

**Authors:** Le Zhang, Xiulan Su

**Affiliations:** Clinical Medical Research Center of the Affiliated Hospital, Inner Mongolia Medical University, 1 Tong Dao Street, Huimin District, 010050 Hohhot, Inner Mongolia China

**Keywords:** Acute myeloid leukemia (AML), N6-methyladenosine (m^6^A), Bioactive peptide (BP), ALKBH5, MLST8, EIF4EBP1

## Abstract

**Purpose:**

N6-methyladenosine (m^6^A), the most prevalent mRNA modification, plays an essential role in tumorigenesis. Notably, increasing interest has been directed to bioactive peptides (BPs) with antitumor activities. Here, we set out to investigate the potential of the BP-regulated ALKBH5/MLST8/EIF4EBP1 axis on prevention and treatment of acute myeloid leukemia (AML).

**Methods:**

The biological effects of BP on AML cells were detected by MTT and ApoLive-Glo™ multiplex assays. The role of BP in tumor growth was determined by a subcutaneous xenograft model. The ALKBH5/MLST8/EIF4EBP1 axis was identified as a potential BP target in AML via methylated RNA immunoprecipitation sequencing (MeRIP-seq) combined with RNA sequencing (RNA-seq). Western blot, RT-qPCR, MeRIP-qPCR, dual-luciferase reporter and RNA stability assays were performed to validate the function and mode of action of the BP-regulated ALKBH5/MLST8/EIF4EBP1 axis. The clinical relevance of the BP-regulated ALKBH5/MLST8/EIF4EBP1 axis in AML was confirmed by TCGA data analysis.

**Results:**

We found that BP can inhibit AML cell proliferation and promote apoptosis *in vitro*, and repress AML tumor growth *in vivo*. Mechanistically, we found that BP downregulated ALKBH5 expression, which in turn repressed m^6^A demethylation of MLST8 and EIF4EBP1 mRNAs. Reduction of the m^6^A levels of MLST8 and EIF4EBP1 facilitated MLST8 and EIF4EBP1 mRNA decay, resulting in inhibition of AML cell proliferation. Furthermore, we found that the BP-regulated ALKBH5/MLST8/EIF4EBP1 axis closely correlates with AML patient prognosis.

**Conclusions:**

Our data indicate that BP can inhibit acute myeloid leukemia cell proliferation by downregulating ALKBH5-mediated m^6^A demethylation of EIF4EBP1 and MLST8 mRNAs, which may have potential to prevent and treat this disease.

**Supplementary Information:**

The online version contains supplementary material available at 10.1007/s13402-022-00666-9.

## Introduction

Cancer continues to be an enormous public health issue worldwide. The International Agency for Research on Cancer estimated that there will be approximately 437,033 new cases of leukemia worldwide in 2018, resulting in 309,006 deaths [1]. The American Cancer Society estimated that 61,090 new cancer cases and 23,660 cancer deaths were projected to occur in the United States in 2021 [2]. Acute myeloid leukemia (AML), a major type of leukemia, is an aggressive, rare and heterogeneous neoplasm [3]. The current first-line treatment for AML is still largely based on combination chemotherapy and stem cell transplantation [4]. Although breakthroughs in AML treatment have been made, the overall survival is still less than 30% for adults and 60% for children [5]. Current data suggest that drug resistance is a major issue that urgently needs to be overcome [3, 6].

Bioactive peptides (BPs) are a class of specific protein fragments ranging from 2 to 20 amino acid residues that exhibit various biological activities and may affect health [7]. BPs have attracted increasing attention since they have been found to exert antioxidant, antihypertensive, anti-inflammatory and antitumor activities. Singh and colleagues reported, for example, that soy peptides exhibit higher antioxidant activities than intact proteins [8]. Anti-hypertensive peptides are inhibitors of ACE that are essential for the regulation of blood pressure. Cicero et al. found that the bioactive peptides IPP and VPP could reduce systolic blood pressure in European subjects [9]. Marine peptides [10], fermented milk-derived peptides [11] and velvet antler protein-derived peptides [12] have shown anti-inflammatory activities. Notably, increasing interest has been directed to BPs with antitumor activities. It has been reported that BPs and depsipeptides derived from marine animals may exhibit cytotoxic effects on several human cancer-derived cell lines. Current data suggest that BPs and depsipeptides may potentially be used for the prevention and treatment of cancer [13]. Chomdao et al. found that the cell membrane-induced folding of SVS-1 BP into an amphiphilic β-hairpin, which could interpolate and disrupt the membranes of cancer cells, may lead to cell death [14]. Antimicrobial peptides, such as defensins, lactoferricin B, cecropins A and B and magainin ii (MG2), have been reported to serve as biological and immunotherapeutic agents against cancer [15]. Another study showed that anion exchanger 2 (AE2)-targeting peptide may simultaneously promote Treg cell apoptosis and enhance effector T-cell functions in B-cell malignancies [16]. The BP applied in this research was isolated from goat liver and were characterized as unique polypeptides with various biological activities. Our previous research revealed that BP can effectively inhibit cell proliferation in gastric cancer [17] and colorectal cancer [18]. As yet, however, the potential role of BP in the prevention and treatment of AML remains elusive.

N6-methyladenosine (m^6^A), methylation of N6 on adenosine, is the most prevalent modification of mRNA [19]. This reversible modification is determined by m^6^A readers, writers and erasers [20]. Two m^6^A demethylases, fat mass and obesity-associated protein (FTO) and AlkB homolog 5 (ALKBH5), have been identified as m^6^A erasers that catalyse m^6^A demethylation [21]. ALKBH5, the second identified m6A demethylase, has been found to play a critical role in various cancer-related characteristics, such as autophagy [22], stem cell self-renewal [23, 24], radioresistance [25] and anti–PD-1 therapy response [26]. Several additional studies have uncovered a regulatory function of ALKBH5 in cancer cell proliferation, invasion and metastasis [27–29]. Other studies have indicated that its functional role in leukemia is limited. Here, we report that BP can inhibit AML cell proliferation and promote apoptosis *in vitro* and repress AML tumor growth *in vivo*. Mechanistically, we found that BP can repress m6A demethylation of MLST8/EIF4EBP1 mRNA by downregulating ALKBH5. ALKBH5 reduced the m6A levels of MLST8 and EIF4EBP1, resulting in reduced mRNA stability. Moreover, we provide evidence that the BP-regulated ALKBH5/MLST8/EIF4EBP1 axis may play an essential role in the prediction of clinical outcome. Collectively, our study provides new insights into the development of potential therapeutic strategies for AML.

## Materials and methods

### Cells and culture conditions

MV4-11, HL-60 and HEK293T cell lines were obtained from the American Type Culture Collection (ATCC). All cells were cultured according to the instructions from the ATCC. The leukemia cell lines were grown in RPMI-1640 medium supplemented with 10% FBS (Gibco), 100 U/ml penicillin/streptomycin (Invitrogen) and 2 mM L-glutamine (Invitrogen). HEK293T cells were grown in DMEM (Invitrogen) supplemented with 10% FBS (Gibco) and 100 U/ml penicillin/streptomycin (Invitrogen).

### Xenograft mouse model

All animal procedures and studies were approved by the ethics committee of the Inner Mongolia Medical University. Five- to six-week-old female CB17 SCID mice were purchased from Beijing Weitonglihua Company (Beijing, China). HL-60 cells (1 × 10^7^) suspended in 0.1 ml PBS containing 50% Matrigel were subcutaneously injected into the flanks of the mice. When tumor sizes reached 200 mm^3^, the mice were randomly distributed into four groups with the indicated dosages of saline, cytarabine and BP alone or in combination. For BP injections, the solution was delivered intraperitoneally at 106 µg/kg body weight for the first 8 consecutive days. For cytarabine injections, the solution was delivered intraperitoneally at 100 mg/kg body weight three times (once every three days). The combination group was administered intraperitoneally three times (once every three days) with the same dosages as described above. The control group was treated with an equivalent amount of saline. All xenograft tumors were allowed to reach an endpoint volume of 1200 mm^3^.

### Plasmids and transfection

Wild-type ALKBH5, H204A mutant ALKBH5, MLST8 and EIF4EBP1 expression plasmids were generated by PCR subcloning of the human ALKBH5 coding sequence into pcDNA3.1 plasmids. DNA fragments of MLST8-3’UTR and EIF4EBP1-3’UTR containing wild-type m^6^A motifs were directly synthesized and inserted downstream of firefly luciferase of a pMIR-Report vector. HEK293T cells were transfected with Lipofectamine 3000 (Thermo Scientific). Ingenio® Electroporation Kits and Solutions were used for electroporation of DNA into MV4-11 and HL-60 cells using Bio-Rad Gene Pulser® electroporation instruments according to the manufacturer’s instructions.

### Apoptosis, viability and proliferation assays

Cells were collected and seeded at the requested concentrations and then treated with the corresponding drugs for a period of 24 h, after which apoptosis and cell viability assays were carried out using an ApoLive-Glo Multiplex Assay Kit (Promega) according to the manufacturer’s instructions. For the proliferation assay, 5000–10,000 cells/well were seeded in 96-well plates in triplicate and then treated with the corresponding drugs for 24, 48, 72 and 96 h. Cell proliferation was assessed by MTT (Promega) following the manufacturer’s instructions. Optical densities (OD) were detected at 570 nm using a SpectraMax i3X Multimode Detection Platform of Molecular Devices once per day for 5 days.

### Western blot analysis

Proteins were separated on denaturing SDS-PAGE gels and transferred onto PVDF membranes. Next, the membranes were blocked with 5% non-fat milk for 1 h at room temperature and incubated overnight at 4 °C with anti-ALKBH5 (ab195377, Abcam), anti-EIF4EBP1 (ab32024, Abcam), anti-MLST8 (ab228832, Abcam), anti-Cleaved caspase 8 (9496, CST), anti-Pro caspase 8 (ab108333, Abcam) and anti-P84 (10920-1-AP, Proteintech) antibodies. Then, the membranes were washed and incubated for 1 h with an HRP-conjugated secondary antibody. An ECL chemiluminescence system (Tanon-4800 Multi) was employed for the detection of proteins.

### RNA extraction and RT-qPCR

Total RNA was isolated using TRIzol (Invitrogen) following the manufacturer’s protocols. First-strand cDNA was synthesized using the GoScript™ Reverse Transcription System (Promega). RT-qPCR analysis was performed using SYBR Green GoTaq® qPCR Master Mix (Promega) in an AB 7500 Real-time PCR Instrument (Applied Biosystems). All reactions were performed in triplicate, and *Gapdh* or *Actin* were used as endogenous controls. The following RT-PCR primers were used:

ALKBH5: 5’- CGGCGAAGGCTACACTTACG − 3’; 5’- CCACCAGCTTTTGGATCACCA − 3’.

MLST8: 5’- ATCCGCATGTATGATCTCAACTC − 3’; 5’- CCACAGACGCGATGTTCTTG − 3’.

EIF4EBP1: 5’- CCTGATGGAGTGTCGGAACT − 3’; 5’- CCGCTTATCTTCTGGGCTATTG − 3’.

### m^6^A-seq assay

m^6^A-seq analysis was performed according to a standard method previously described [23, 28, 31]. Polyadenylated RNA was fragmented and incubated with a specific anti-m^6^A antibody (Synaptic Systems) for 2 h at 4 °C. Captured RNA was washed and then eluted and purified using an RNeasy Mini Kit (QIAGEN). Library preparation was performed using a SMARTer Stranded Total RNA-Seq Kit v2 - Pico Input Mammalian (TaKaRa-Clontech). The samples were sequenced using a NextSeq 500 High Output Mode 75 cycles kit (Illumina).

### m^6^A quantification

Changes in global m^6^A levels in mRNAs were measured using an EpiQuik m^6^A RNA Methylation Quantification Kit (Epigentek) according to the manufacturer’s instructions. Briefly, total RNA was bound to strip wells using RNA high-binding solution. m^6^A was detected using capture and detection antibodies. The detected signals were enhanced and then quantified colorimetrically by reading the absorbance using a microplate spectrophotometer. The amount of m^6^A is proportional to the OD intensity measured.

### MeRIP-qPCR

MeRIP was performed according to a previously reported protocol [30]. Polyadenylated RNA was incubated with an anti-m^6^A antibody (Synaptic Systems) in 500 µl IP buffer (150 mM NaCl, 0.1% NP-40, 10 mM Tris, pH 7.4, 100 U RNase inhibitor) for 2 h at 4 °C. Next, Dynabeads® Protein A (Thermo Fisher Scientific) was added to the mixture and rotated for another 2 h at 4 °C. The captured RNA was washed 4 times, eluted twice with 50 µl m^6^A-elution buffer (IP buffer, 6.7 mM m^6^A, 30 U RNase inhibitor), incubated and shaken at 4 °C for 1 h and precipitated with 5 mg glycogen and one-tenth volumes of 3 M sodium acetate in 2.5 volumes of 100% ethanol at -80 °C overnight. Gene-specific m^6^A enrichment was assessed by qPCR.

### Luciferase reporter assay

Cells seeded in 12-well plates were cotransfected with 200 ng pcDNA3.1- ALKBH5-WT/H204A, 20 ng pRL-TK (Renilla luciferase control reporter vector) and 300 ng pMIR-Report luciferase vector (Promega) fused with the wild-type EIF4EBP1-3’UTR and MLST8-3’UTR. 24 h after transfection, cells were treated with BP for a period of 24 h. Then, the relative luciferase activities were calculated using a Dual-luciferase Reporter Assay System (Promega). Each experiment was repeated in trice.

### RNA stability assay

MV4-11 and HL-60 cells seeded in 6-well plates were treated with BP for a period of 48 h, after which 5 µg/ml actinomycin D was added at 6 h, 3 h and 0 h before cell scraping and collection. Total RNA was isolated using an RNeasy kit (QIAGEN) under conditions recommended by the manufacturer. The relative levels of target mRNA were determined by RT-PCR. The degradation rate of target mRNA was estimated according to a previously published method[19].

### Immunohistochemistry

After deparaffinization and rehydration, tumor sections were immunostained using an anti-Ki67 monoclonal antibody. All images were captured at 20x magnification. The procedure was carried out according a previously published method [31].

### Statistical analysis

All statistical analyses were performed using the SPSS 20.0 software package (IBM, Chicago, IL, USA). The significance of the differences was assessed using the two-tailed Student’s t-test or a chi-squared test, as appropriate. Data are presented as the mean ± SEM derived from three independent experiments. Significant *p*-values are indicated as follows: not significant (ns); *p*-value < 0.05 (*); *p*-value < 0.01 (**); *p*-value < 0.001 (***).

## Results

### BP suppresses cell proliferation and promotes apoptosis *in vitro*

**Fig. 1:**
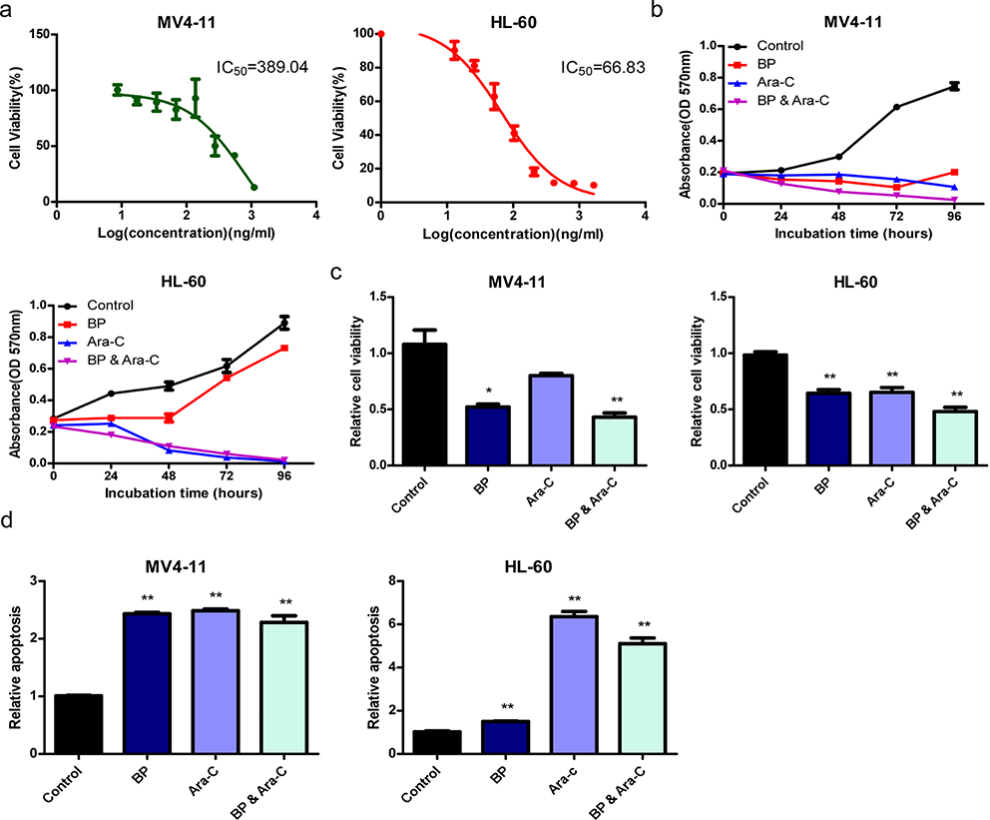
**BP suppresses cell proliferation and promotes apoptosis** ***in vitro *a** MV4-11 and HL-60 cells were seeded in 96-well plates for 24 h and then treated with different BP concentrations. After 48 h, the absorbance at 450 nm was measured, and IC50 values were calculated from the percent inhibition values of cell viability. **b** Cell growth activity was measured using an MTT assay. MV4-11 and HL-60 cells were treated with saline, BP (389.04 ng/ml for MV4-11; 66.83 ng/ml for HL-60), cytarabine (3 mM for MV4-11; and 2 mM for HL-60), or combinations of BP and cytarabine for 24, 48, 72 and 96 h. **c, d** Cell viability and apoptosis were detected using an ApoLive-Glo™ Multiplex assay. MV4-11 and HL-60 cells were treated with saline, BP (389.04 ng/ml for MV4-11; 66.83 ng/ml for HL-60), cytarabine (3 mM for MV4-11; and 2 mM for HL-60), or combinations of BP and cytarabine for 24 h. The results are expressed as the mean ± SD (n = 3), *, *p* < 0.05, **, *p* < 0.01.

The IC50 of BP on AML cells was evaluated using a CCK-8 assay to determine the proper concentration required for 50% inhibition (IC50). The IC50 values of BP were 389.04 ng/ml and 66.83 ng/ml for MV4-11 and HL-60 cells, respectively (Fig. 1a). To examine the biological effects of BP, we monitored cell proliferation, viability, and apoptosis under BP treatment in different human leukemia cell lines, including MV4-11 and HL-60. First, we employed a MTT assay to investigate the effect of BP treatment on AML cell proliferation. As shown in Fig. 1b, we found that the BP group, cytarabine group and BP and cytarabine combination group exhibited lower proliferation rates than the control group. Significantly, the BP and cytarabine combination group showed the lowest growth rate (Fig. 1b). For further investigation, we performed an ApoLive-Glo™ Multiplex assay to assess the viability and caspase activity of AML cells under different treatment conditions. Similarly, we found that compared to the control group, treatment with BP or cytarabine decreased cell viability and promoted apoptosis. As expected, the combination of BP and cytarabine led to the lowest cell viability (Fig. 1c, d). Taken together, these results suggest that BP suppresses cell proliferation and promote apoptosis *in vitro*.

### BP significantly suppresses tumor growth *in vivo*

**Fig. 2:**
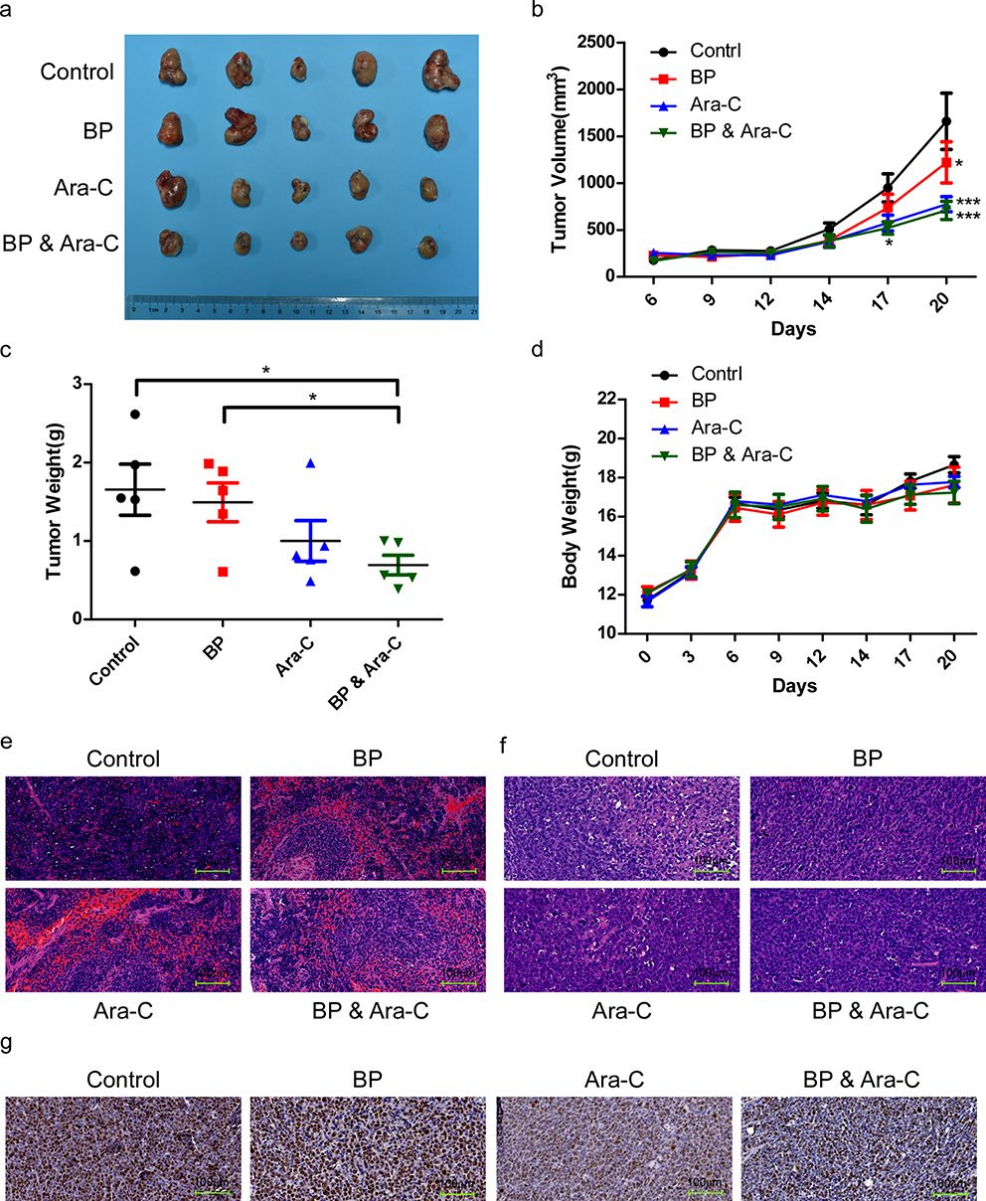
**BP suppresses tumor growth *in vivo *a** When tumor sizes reached 200 mm^3^, mice were randomly distributed into four groups with the indicated treatments (BP: 106 µg/kg for the first 8 consecutive days; Ara-C: 100 mg/kg once every three days for three times; BP & Ara-C: delivered intraperitoneally once every three days for three times using the same dosages as described above). Representative images of subcutaneous tumors isolated from mice at the experimental endpoint are shown. **b** Tumor volumes of the indicated treatment xenografts. Data are presented as the mean ± SEM (n = 5). **c** Tumor weight of the indicated treated xenografts at the experimental endpoint. The tumor weight is expressed as the mean ± SEM (n = 5). **d** Body weights after the indicated treatments. **e, f** Haematoxylin and eosin staining of spleens and tumors from the indicated treatment groups. **g** Ki-67 immunohistochemistry of tumors from the indicated treatment groups. All images were captured at 20x magnification.

We next employed a xenograft model of HL-60 cells to examine the effect of BP on tumor growth *in vivo*. We found that BP treatment significantly (*p* < 0.05; log-rank test) slowed down tumor growth in tumor-bearing mice (Fig. 2a). Across multiple time points, a significantly lower (26.4% lower at the endpoint) tumor volume was detected in mice treated with BP compared to that in the control group (Fig. 2b). Consistently, at the experimental endpoint, mice treated with BP carried tumors with a lower weight than those formed in the control group (Fig. 2c). Furthermore, we explored the benefit of combining BP with cytarabine in the HL-60 xenograft model. As shown in Fig. 2a, c, we found that tumors in the combination group were significantly smaller and weighted less those in the single BP or cytarabine treatment group. At each time point, there was no significant body weight loss, which indicates that all treatments were well tolerated (Fig. 2d). Then, we employed H&E staining to assess the histologies of the respective spleens (Fig. 2e). We found that compared to the control group, all treatments relieved the disruption of the spleen structure, including white pulp atrophy and boundaries separating white and red pulp blur. In addition, the histologies of the subcutaneous tumors were confirmed by H&E staining, with many tumor cells observed in the tumor mass (Fig. 2f). By utilizing immunohistochemistry, we found that the proportion of Ki67-positive cells, a marker of cell proliferation, in the BP group was significantly lower than that in the control group, but slightly greater than that in the cytarabine group or the combination of BP and cytarabine group (Fig. 2 g). Collectively, these data indicate that BP can significantly inhibit AML tumor growth *in vivo*.

### Identification of potential BP targets in AML using transcriptome-wide m^6^A-Seq and RNA-Seq

**Fig. 3:**
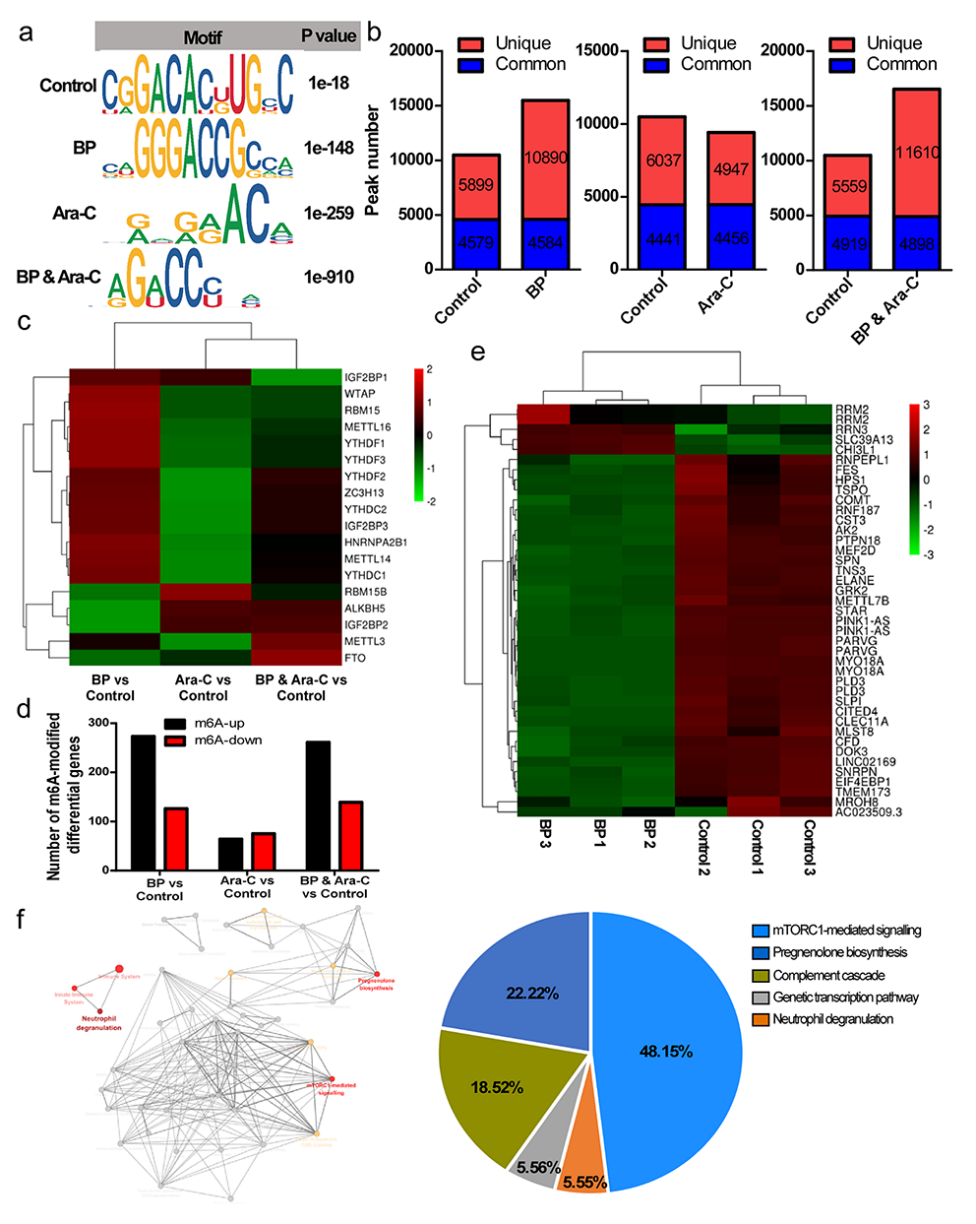
**m** ^**6**^**A Modification and potential targets of BP a** Top consensus m^6^A motif under different treatments. **b** Number of m^6^A peaks identified in m^6^A-seq. **c** The expression of RNA methylation regulators changed under different treatment conditions compared with the control sample according to m^6^A-seq. **d** Number of m^6^A-modified genes under different treatment conditions compared with the control sample according to m^6^A-seq. **e** The leukemia-related gene set filtered by the Molecular Signatures Database (MSigDB) is shown in a heatmap. **f** Results of pathway enrichment analysis performed in ClueGO (plugin for Cytoscape software) shows that mTORC1-mediated signalling, mTOR signalling and TOR signalling are significantly enriched.

Emerging evidence indicates that m^6^A modification is involved in the progression of various cancers. Therefore, we speculate that BP may inhibit leukaemic cell proliferation and induce apoptosis, leading to suppression of leukemia tumorigenesis by regulating mRNA m^6^A enrichment and expression. Using m^6^A sequencing (m^6^A-seq), we mapped the m^6^A methylomes of AML cells under different treatment conditions. We found that the GGACC motif was highly enriched within m^6^A sites of BP-treated AML cells (Fig. 3a). In total, we identified 10,478 m^6^A peaks representing the transcripts of 7684 genes in control cells, 15,474 m^6^A peaks representing the transcripts of 10,369 genes in BP-treated cells, 9403 m^6^A peaks representing the transcripts of 6901 genes in cytarabine-treated cells, and 16,508 m^6^A peaks representing the transcripts of 10,829 genes in combination-treated cells (Fig. 3b; Fig. S1a). Furthermore, m^6^A-seq revealed that the expression of regulators related to RNA methylation changed under different treatment conditions compared with the control samples. Compared to those in the control group, methyltransferases, such as METTL3, METTL14, METTL16, WTAP, RBM15 and ZC3H13, in the BP treatment group were markedly upregulated, while the demethylase ALKBH5 was significantly downregulated. The combination treatment group shared similar changes, while there was no significant difference in the cytarabine group (Fig. 3c; Table S1). Given the significant changes in m^6^A modification, we further explored the target genes affected by BP treatment. A total of 399 genes showed differential methylation modification in the BP group, in which methylation modification of 273 genes was upregulated, while that in 126 genes was downregulated. Similar to the BP group, 261 genes showed upregulated methylation modifications, and 139 genes showed downregulated methylation modifications in the BP and cytarabine combination group. Of the 139 genes with changes in methylation modification in the cytarabine group, 64 exhibited upregulated changes, while 75 were linked with downregulated changes (Fig. 3d). Four quadrant graph analysis revealed that the mRNA m^6^A level increased in the BP treatment group and indicated that genes with upregulated methylation are expected to encompass genuine targets of BP treatment (Fig. S1b). Among genes with upregulated methylation in the BP group, we found that the expression of 5 genes was increased and that of 36 genes was decreased (FC > 2 & *p* < 0.05). We employed the Molecular Signatures Database (MSigDB) to analyse the genes filtered above and found that 26 out of 36 downregulated genes and 3 out of 5 upregulated genes were included in the leukemia-related gene set (Fig. 3e; Table S2). We next investigated the pathways of the differentially expressed genes that were shared by ClueGO (plugin for Cytoscape software) and found that the significantly enriched pathways included mTORC1-mediated signalling, mTOR signalling and TOR signalling (Fig. 3f). Therapeutic targeting of the mTOR pathway as an anticancer strategy for leukemia has been under extensive investigation. mTOR signalling plays a central role in the proliferation and survival of AML and is aberrantly activated in 60% of AML patients [32, 33]. mTOR, which is a serine/threonine kinase, is a major component of different protein complexes, including mTOR complex 1 (mTORC1) and mTOR complex 2 (mTORC2) [34]. mTORC1 controls mRNA translation and protein synthesis through phosphorylation of S6K and 4E-BP [35], whereas mTORC2 mainly mediates signal transduction by regulating AKT kinase [36, 37]. In the BP group, the expression of EIF4EBP1 and MLST8 was significantly decreased. Next, we focused on these two potential ALKBH5 targets for further study.

### BP represses demethylation of m^6^A in MLST8/EIF4EBP1 mRNA by downregulating ALKBH5

**Fig. 4:**
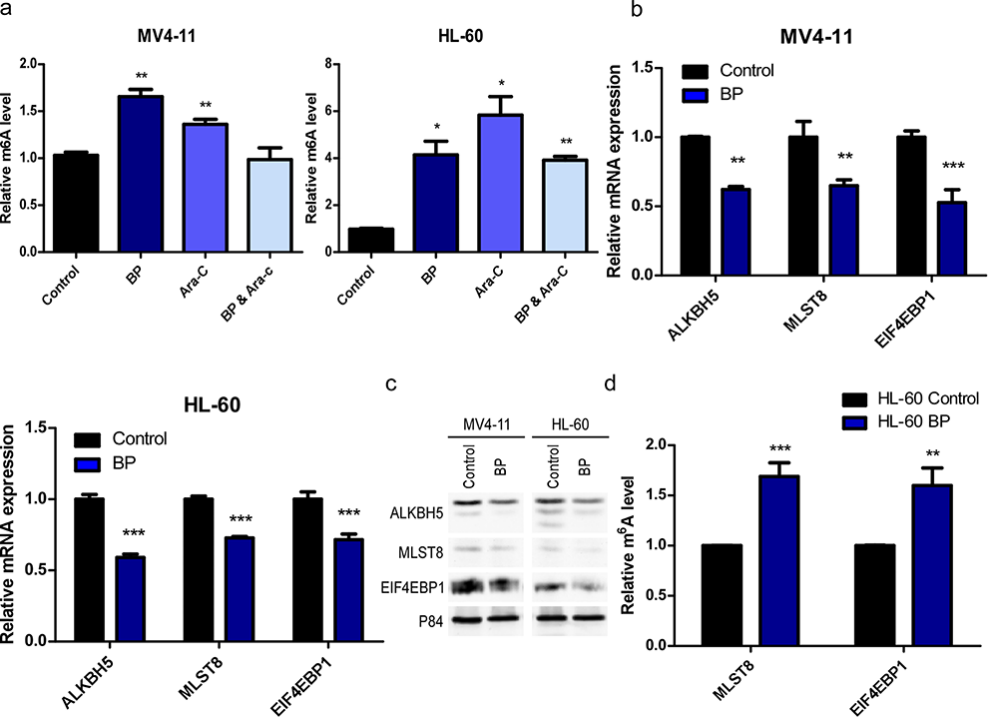
**BP represses demethylation of m**^**6**^**A in MLST8/EIF4EBP1 mRNA a** Total RNA was extracted and changes in global m^6^A levels were measured using an EpiQuik m^6^A RNA Methylation Quantification Kit. **b** RT-qPCR was performed to analyse ALKBH5, MLST8 and EIF4EBP1 mRNA expression under BP treatment. **c** Western blotting was performed to analyse ALKBH5, MLST8 and EIF4EBP1 protein expression under BP treatment. **d** m^6^A RNA immunoprecipitation followed by RT-qPCR was used to determine the levels of MLST8 and EIF4EBP1 m^6^A methylation under BP treatment.

Next, we investigated whether BP treatment increases the m^6^A RNA modification levels in cells. Consistent with the previous m^6^A-seq results, we found that BP treatment significantly increased global m^6^A levels in leukaemic cells (Fig. 4a). Since MLST8/EIF4EBP1 may act directly downstream of ALKBH5 and drive the altered proliferative effects, we set out to investigate the underlying mechanism. First, we confirmed their protein and mRNA levels by Western blot and RT-qPCR analyses, respectively. Compared to control cells, BP-treated MV4-11 and HL-60 cells showed both decreased protein and mRNA levels of ALKBH5, MLST8 and EIF4EBP1 (Fig. 4b, c). Next, we employed methylated RNA immunoprecipitation (Me-RIP) combined with RT-qPCR to determine the levels of MLST8 and EIF4EBP1 m^6^A methylation following BP treatment. We found that the m^6^A levels of MLST8 and EIF4EBP1 in BP-treated cells were significantly increased compared to those in control cells (Fig. 4d). Together, these results indicate that MLST8 and EIF4EBP1 act as direct downstream targets of ALKBH5 in AML.

### BP suppresses ALKBH5-mediated reduction in MLST8/EIF4EBP1 mRNA stability by catalysing m^6^A demethylation

**Fig. 5:**
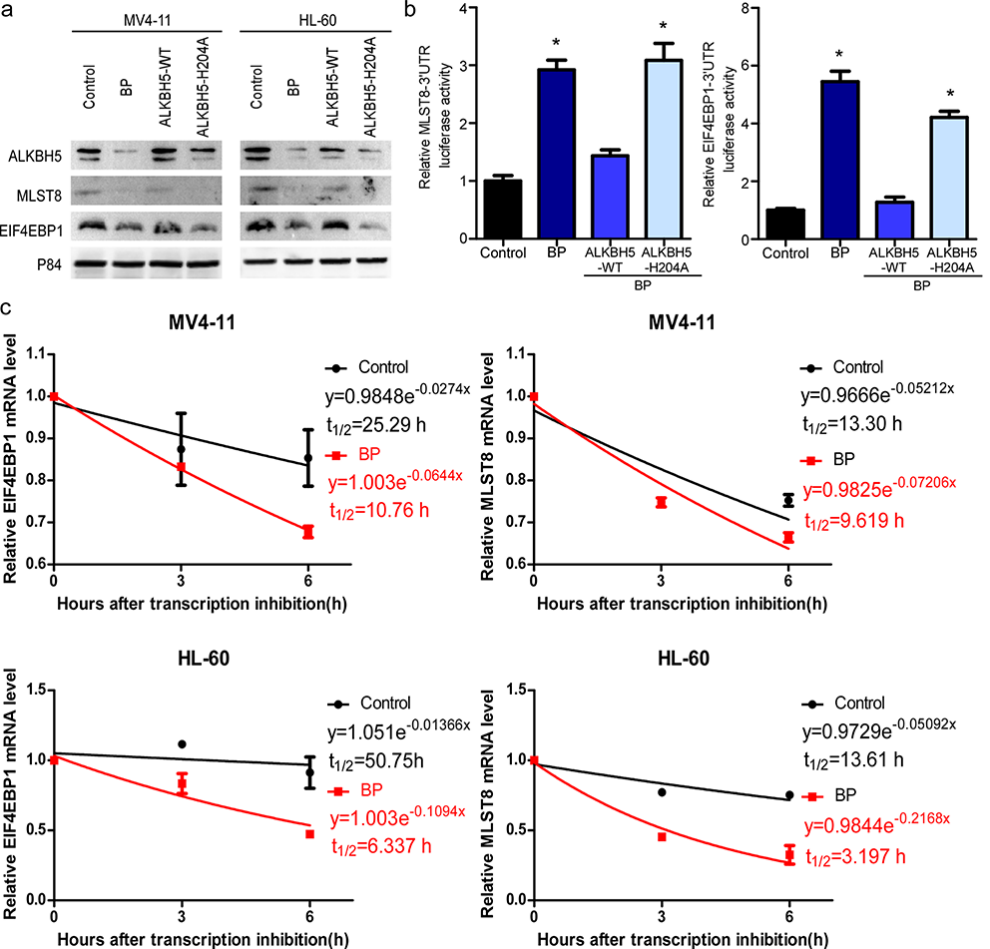
**BP suppresses the ALKBH5-mediated enhancement in the RNA stability of MLST8/EIF4EBP1 by catalysing m**^**6**^**A demethylation a** Protein expression of MLST8 and EIF4EBP1 detected by Western blotting under BP treatment of cells transduced with ALKBH5-WT or ALKBH5-H204A. **b** Relative activities of MSLT8 3’UTR and EIF4EBP1 3’UTR firefly luciferase reporters harbouring either wild-type or mutant (A-to-T mutation) m^6^A sites after cotransfection with ALKBH5, ALKBH5 mutant or control vector into HEK293T cells with or without BP treatment. **c** MV4-11 and HL-60 cells were treated with BP for a period of 48 h and then treated with vehicle or 5 µg/ml actinomycin D for 3 or 6 h. The mRNA half-life (t_1/2_) of MLST8 or EIF4EBP1 was determined by RT-qPCR.

To investigate the importance of the demethylation activity for ALKBH5-induced downregulation of MLST8/EIF4EBP1 expression, we constructed an ALKBH5 mutant H204A that has completely lost its demethylation activity. Next, we employed Western blotting to examine protein expression of MLST8 and EIF4EBP1. We found that overexpression of wild-type ALKBH5, but not mutant H204A, could rescue the expression of MLST8 and EIF4EBP1 in BP-treated cells (Fig. 5a). Sequence analysis revealed that there were three and two matches to the 5′-RRACU-3′ (methylated adenosine residue is underscored) m^6^A consensus sequences within the MLST8 and EIF4EBP1 3′-UTRs, respectively. Next, we constructed m^6^A consensus sequences within the MLST8 and EIF4EBP1 3′-UTRs in the luciferase plasmid and found that compared with the control, BP treatment resulted in constitutively increased luciferase activities of MLST8 and EIF4EBP1. Overexpression of wild-type ALKBH5, but not inactive mutant H204A, could rescue BP-induced enhancement of the luciferase activities of MLST8 and EIF4EBP1(Fig. 5b). We utilized actinomycin D to block de novo RNA synthesis to assess the RNA stability of MLST8 and EIF4EBP1. BP treatment significantly decreased the half-life of MLST8 and EIF4EBP1 mRNA transcripts in AML cells (Fig. 5c). These results indicate that BP may suppress the ALKBH5-mediated reduction in the RNA stability of MLST8/EIF4EBP1 by catalysing m^6^A demethylation.

### BP-mediated inhibition of cell proliferation depends on the ALKBH5/MLST8/EIF4EBP1 axis

**Fig. 6:**
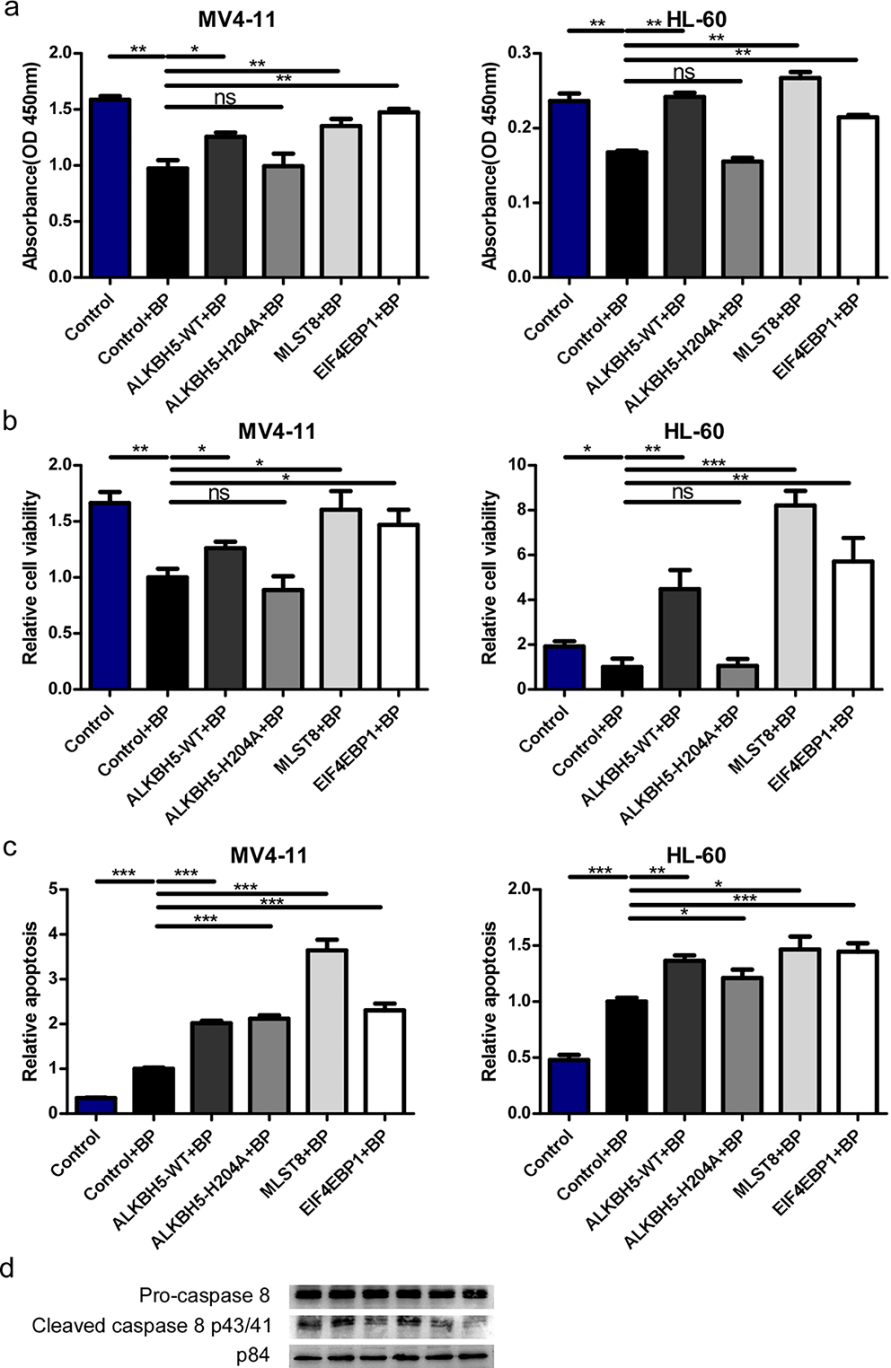
**BP-mediated inhibition of cell proliferation relies on the ALKBH5/MLST8/EIF4EBP1 axis a** MV4-11 and HL-60 cells were transfected with ALKBH5, ALKBH5-H204A, MLST8, EIF4EBP1 or the control vector. After 24 h, MV4-11 and HL-60 cells were treated with or without BP (389.04 ng/ml for MV4-11; 66.83 ng/ml for HL-60) for 48 h followed by the addition of CCK8 for 3 h and measurement of absorbance at 450nm. **b, c** Cell viability and apoptosis detected using an ApoLive-Glo™ Multiplex assay. MV4-11 and HL-60 cells were transfected with ALKBH5, ALKBH5-H204A, MLST8, EIF4EBP1 or a control vector. After 24 h, MV4-11 and HL-60 cells were treated with or without BP (389.04 ng/ml for MV4-11; 66.83 ng/ml for HL-60) for 24 h followed by cell viability and apoptosis detection. The results are expressed as the mean ± SD (n = 7), *, *p* < 0.05, **, *p* < 0.01. **d** Expression levels of apoptosis related genes Pro-caspase 8 and Cleaved-caspase 8 detected by Western blotting.

To further determine whether the inhibitory effect of BP on AML cell proliferation relies on the ALKBH5/MLST8/EIF4EBP1 axis, we performed a rescue experiment. A CCK-8 assay showed that BP treatment resulted in a lower proliferation rate compared to that in the control group. Notably, compared with BP treatment, overexpression of MLST8 and EIF4EBP1 drastically increased cell proliferation. Moreover, overexpression of wild-type ALKBH5, but not mutant H204A, significantly rescued BP-induced inhibition of cell proliferation (Fig. 6a). Next, we performed an ApoLive-Glo™ Multiplex assay to assess the viability and caspase activity of these stable AML cells. In accordance with the increased cell proliferation, we found that re-expression of ALKBH5/MLST8/EIF4EBP1 led to rescue of the reduced viability, while mutant ALKBH5 H204A had lost this regulatory function (Fig. 6b). Interestingly, we found that BP treatment led to increased apoptosis, while reintroduction of ALKBH5-WT/ALKBH5-H204A/MLST8/EIF4EBP1 upregulated the BP-induced promotion of apoptosis (Fig. 6c). We also assessed the expression levels of apoptosis related genes including Pro-caspase 8 and Cleaved-caspase 8 and found that BP treatment resulted in upregulation of Cleaved-caspase 8 p43/p41compared to the control group. Overexpression of ALKBH5-WT/MLST8/EIF4EBP1 could reverse the increased Cleaved-caspase 8 level rather than mutant ALKBH5 H204A (Fig. 6d). These data suggest that BP-mediated inhibition of cell proliferation depends on the ALKBH5/MLST8/EIF4EBP1 axis.

### Clinical relevance of the BP-regulated ALKBH5/MLST8/EIF4EBP1 axis in AML

**Fig. 7:**
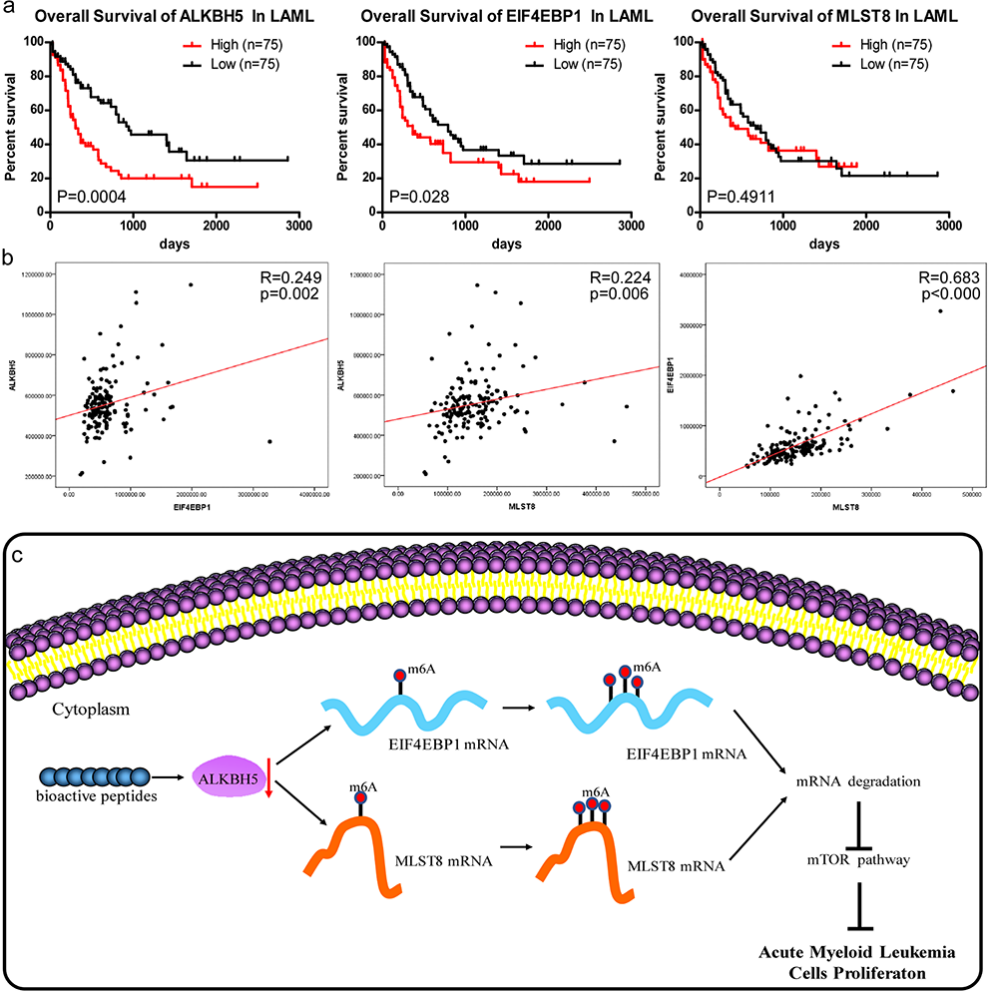
**Clinical relevance of the BP-regulated ALKBH5/MLST8/EIF4EBP1 axis in AML a** Overall survival analysed using the TCGA database based on expression levels of ALKBH5, MLST8 and EIF4EBP1. **b** Pearson correlation between ALKBH5 and MLST8 or EIF4EBP1 mRNA expression in the TCGA dataset. **c** Schematic representation of the underlying mechanism of BP inhibition of acute myeloid leukemia cell proliferation. Bs downregulate ALKBH5 and, thereby, decrease m^6^A demethylation of EIF4EBP1 and MLST8 mRNA, contributing to reduced EIF4EBP1 and MLST8 mRNA stability.

To investigate whether the BP-regulated ALKBH5/MLST8/EIF4EBP1 axis has clinical relevance in AML patients, we analysed ALKBH5/MLST8/EIF4EBP1 expression profiles of AML patients for whom overall survival (OS) data were available in the TCGA dataset. We found that a lower expression was positively correlated with better OS among these patients. The median survival times of patients with a lower expression of ALKBH5, MLST8 and EIF4EBP1 were 950 days, 650 days and 700 days, respectively, whereas thoseof patients expressing higher levels were 334 days, 400 days and 350 days, respectively (Fig. 7a). Next, we examined expression correlation among ALKBH5, MLST8 and EIF4EBP1 in the TCGA dataset of AML patient samples. We found a statistically positive correlation between the expression levels of ALKBH5 and EIF4EBP1 or MLST8 (Pearson’s r = 0.249, *p* = 0.002; Pearson’s r = 0.224, *p* = 0.006) (Fig. 7b). These data suggest a critical role of the ALKBH5/MLST8/EIF4EBP1 axis in the prediction of clinical outcome and highlight the potential of the ALKBH5/MLST8/EIF4EBP1 axis as a molecular target in AML. Overall, our data indicate that BP-mediated downregulation of ALKBH5 leads to decreased m^6^A demethylation of EIF4EBP1 and MLST8 mRNA and, thereby, reduced EIF4EBP1 and MLST8 RNA stability, contributing to inhibition of AML cell proliferation (Fig. 7c).

## Discussion

In the present study, we revealed a key role of BP in AML progression. Specifically, we found that BP can inhibit AML cell proliferation and promote apoptosis *in vitro*, and reduce tumor growth *in vivo*. Further transcriptome-wide m^6^A-seq assays revealed that the expression of the demethylase ALKBH5 in the BP treatment group was significantly downregulated compared to that in the control group. By combining RNA-Seq and pathway analyses, we identified MLST8 and EIF4EBP1 as potential BP targets in AML. Subsequent validation studies showed that BP can repress demethylation of m^6^A in MLST8/EIF4EBP1 mRNA by downregulating ALKBH5. ALKBH5 reduced the m^6^A levels of MLST8 and EIF4EBP1, resulting in reduced RNA stability. Subsequent rescue studies suggested that BP-mediated inhibition of cell proliferation depends on the ALKBH5/MLST8/EIF4EBP1 axis. Interestingly, we found that re-expression of ALKBH5/MLST8/EIF4EBP1 rescued the reduced viability and, unexpectedly, promoted cell apoptosis. By using the ApoLive-Glo™ multiplex assay caspase-3/7 activation can be detected, which is a key apoptosis biomarker. Huang et al. reported that activated caspase 3 may regulate tumor cell repopulation [38]. This notion supports the function of re-expressing ALKBH5/MLST8/EIF4EBP1 to simultaneously promote cell viability and apoptosis. Moreover, we provide data indicating that the BP-regulated ALKBH5/MLST8/EIF4EBP1 axis may be used to predict clinical outcome.

ALKBH5, a second RNA demethylase, has been reported to play broad and critical roles in fundamental biological processes [21, 39, 40]. Although ALKBH5 has also been implicated in some types of cancers [29, 41–43], its AML-associated function and mechanism have remained unclear. Here, we report that decreased ALKBH5 expression represses the demethylation of m^6^A in MLST8/EIF4EBP1 mRNA, leading to downregulation of these two genes at both the RNA and protein levels, thereby suppressing cell proliferation and promoting apoptosis.

The mTOR pathway exerts its functions through distinct protein complexes, i.e., mTORC1 and mTORC2 [44]. Eukaryotic translation initiation factor 4E binding protein 1 (4EBP1) is a downstream effector of mTORC1 that can be phosphorylated by mTORC1 to participate in various biological processes, and its dysregulation may play an essential role in tumorigenesis by regulating the synthesis of mTORC1 downstream factors [45]. MLST8 is a common member of both mTORC1 and mTORC2 [46]. An increasing number of studies has shown that mTOR is a major regulator of cell survival, growth, protein synthesis and metabolism in tumors [47]. It has been reported that mTOR is aberrantly overactivated in more than 70% of cancers [45]. Especially in AML, 50–80% of the cases show a constitutively activated mTOR pathway, which may induce malignant cell proliferation and drive chemotherapy resistance [48]. Considering the fundamental role of mTOR in tumor progression, it may serve as an important therapeutic target. In fact, two mTOR inhibitors, everolimus and temsirolimus, have been approved by the FDA for renal cell carcinoma treatment [47]. In addition, several mTOR inhibitors are currently undergoing preclinical studies and clinical trials [47] (ICSN3250, OSU-53, AZD8055, LY3023414, everolimus, rapamycin). Unfortunately, due to the multiplicity of mechanisms involved, crosstalk in signalling networks and drug resistance acquisition, mTOR inhibitors exhibit less efficacies than originally expected [47, 49]. Thus, searching for alternative candidates to target mTOR seems to be a good choice to achieve a better clinical benefit. We found that BP can repress the mTOR pathway by downregulating MLST8 and EIF4EBP1 to inhibit AML cell proliferation and *in vivo* tumor growth. Additional evidence has shown that BPs have the advantages of high specificity, few side effects and less drug resistance [50, 51]. Therefore, BP may be ideal candidates to target mTOR, thereby paving the way for developing more efficacious anticancer treatment options.

In addition to applying BP in monotherapy, we conducted preclinical studies combining BP with the chemotherapeutic drug cytarabine. We found that combination treatment resulted in a lower proliferation rate than single BP or cytarabine treatment using a MTT assay. An ApoLive-Glo™ multiplex assay showed that the combination of BP and cytarabine significantly decreased AML cell viability compared to BP or cytarabine alone. *In vivo*, we found that combining BP and cytarabine markedly inhibited tumor growth and prolonged the overall survival time of tumor-bearing mice. Our study may provide new insight into combination therapies with an improved anticancer effect in AML.

In conclusion, we found that BP can inhibit AML cell proliferation and tumor growth by downregulating ALKBH5 to repress m^6^A demethylation of MLST8/EIF4EBP1 mRNA. Our study also provides a proof-of-concept for a therapeutic strategy in which the combination of BP and cytarabine may yield improved anticancer efficacy.

## Electronic Supplementary Material

Below is the link to the electronic supplementary material.

Supplementary Material 1

## Data Availability

All data generated or analyzed during this study are included in this published article [and its additional files].

## Abbreviations

m^6^A: N6-methyladenosine.
AML: acute myeloid leukemia.
BP: bioactive peptide.
MG2: magainin II.
AE2: anion exchanger 2.
METTL3: methyltransferase-like 3.
WTAP: Wilms tumor 1-associated protein.
FTO: fat mass and obesity-associated protein.
ALKBH5: AlkB homolog 5.
CSC: cancer stem cell.
HIF-1α: hypoxia-inducible factor 1α.
Me-RIP: methylated RNA immunoprecipitation.
4EBP1: eukaryotic translation initiation factor 4E-binding protein 1.
