# Supplementary material for: Bioactive peptide inhibits acute myeloid leukemia cell proliferation by downregulating ALKBH5-mediated m6A demethylation of EIF4EBP1 and MLST8 mRNA
Source: Cell Oncol (Dordr). 2022 May 17;45(3):355–65. doi: 10.1007/s13402-022-00666-9 (PMC9187541; doi:10.1007/s13402-022-00666-9)
Supplement: Supplementary file 1 — Supplementary Material 1 [file 13402_2022_666_MOESM1_ESM.docx]

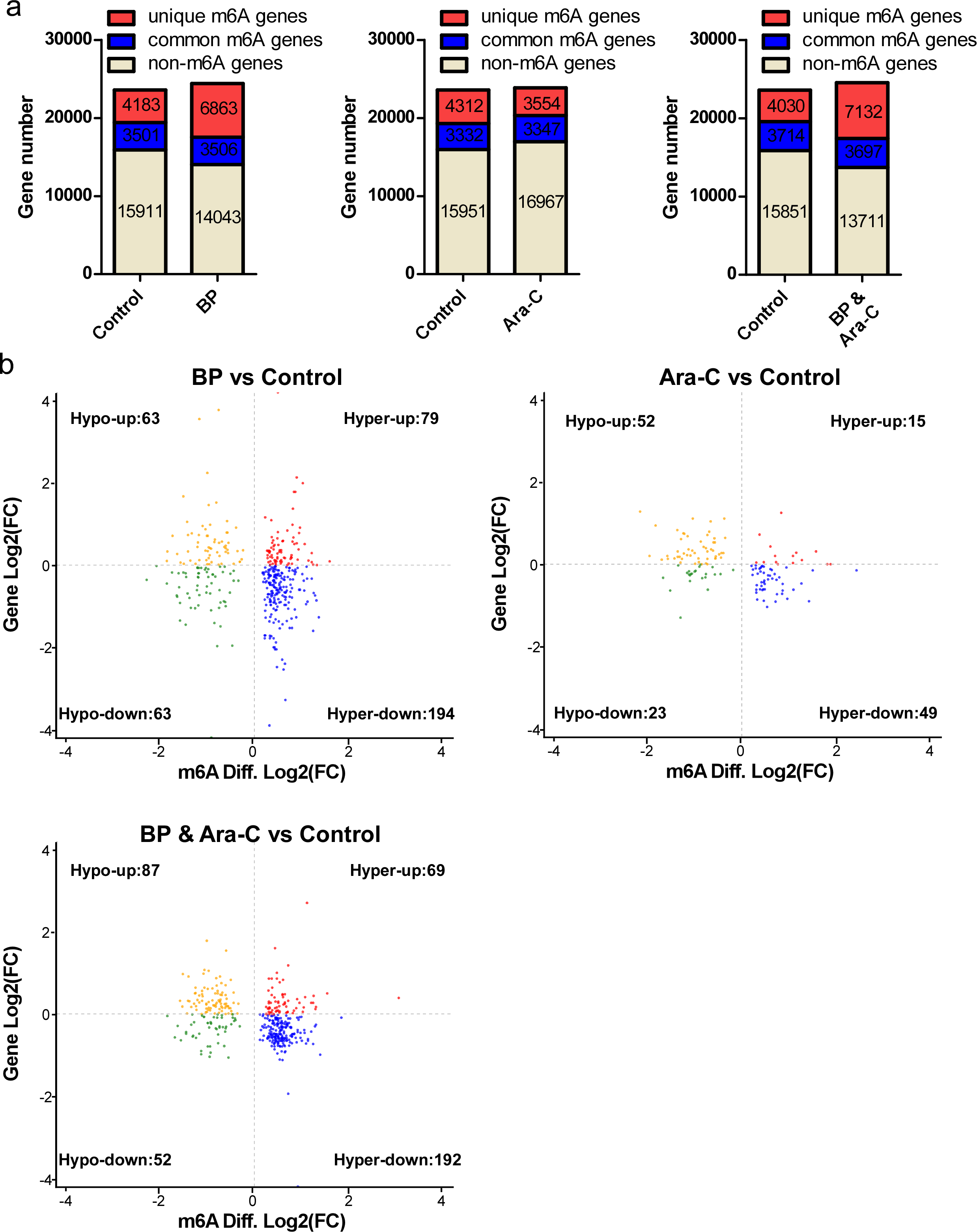


**Fig. S1. m6A Modification map.**

**a** Number of m^6^A-modified genes, including common m^6^A genes (containing at least one common m^6^A peak), unique m^6^A genes (containing no common m^6^A peaks) and non-m^6^A genes identified by m^6^A-seq. **b** Distribution of genes with a significant change in both m^6^A level and expression level in different treatment groups compared to the control group.

**Table S1. m^6^A methylation-related gene expression in different groups**

|  | gene | Function | Description | ACBP vs C（fc） | Acr vs C（fc） | A_A vs C（fc） |
| --- | --- | --- | --- | --- | --- | --- |
| Writers | METTL3 |  | methyltransferase like 3 | 1.16 | 1.09 | 1.2 |
|  | METTL14 |  | methyltransferase like 14 | 1.65 | 1.07 | 1.36 |
|  | METTL16 |  | methyltransferase like 16 | 2.7 | 0.77 | 1 |
|  | WTAP |  | WT1 associated protein | 1.62 | 1.08 | 1.12 |
|  | RBM15 |  | RNA binding motif protein 15 | 1.43 | 1.13 | 1.15 |
|  | RBM15B |  | RNA binding motif protein 15B | 0.82 | 1.09 | 0.91 |
|  | ZC3H13 |  | zinc finger CCCH-type containing 13 | 1.41 | 1 | 1.28 |
| [Erasers](javascript:;) | FTO |  | FTO, alpha-ketoglutarate dependent dioxygenase | 0.92 | 1.03 | 1.49 |
|  | ALKBH5 |  | alkB homolog 5, RNA demethylase | 0.52 | 0.98 | 0.95 |
| Readers | YTHDF1 | Translation | YTH N6-methyladenosine RNA binding protein 1 | 1.28 | 1.18 | 1.21 |
|  | YTHDF2 | decay | YTH N6-methyladenosine RNA binding protein 2 | 1.65 | 1.02 | 1.5 |
|  | YTHDF3 | Translation/ Stability | YTH N6-methyladenosine RNA binding protein 3 | 1.61 | 0.71 | 0.88 |
|  | YTHDC1 | splicing | YTH domain containing 1 | 1.59 | 1.02 | 1.33 |
|  | YTHDC2 | Translation/ Stability | YTH domain containing 2 | 1.41 | 0.95 | 1.26 |
|  | HNRNPA2B1 | miRNA biogenesis | heterogeneous nuclear ribonucleoprotein A2/B1 | 1.3 | 0.84 | 1.05 |
|  | IGF2BP1 | Translation | insulin like growth factor 2 mRNA binding protein 1 | 1.86 | 1.49 | 0.43 |
|  | IGF2BP2 | Translation | insulin like growth factor 2 mRNA binding protein 2 | 0.46 | 0.87 | 0.9 |
|  | IGF2BP3 | Translation | insulin like growth factor 2 mRNA binding protein 3 | 3.38 | 0.93 | 2.34 |

**Table S2. Leukemia-Related Gene Expression Profile in BP group**

| gene | Description | log2(fc) | pval | qval | regulation |
| --- | --- | --- | --- | --- | --- |
| STAR | steroidogenic acute regulatory protein | -3.28 | 0 | 0 | down |
| CLEC11A | C-type lectin domain containing 11A | -2.48 | 0 | 0 | down |
| SLPI | secretory leukocyte peptidase inhibitor | -2.4 | 0 | 0 | down |
| TMEM173 | transmembrane protein 173 | -2.3 | 0 | 0 | down |
| PLD3 | phospholipase D family member 3 | -2.05 | 0 | 0 | down |
| MYO18A | myosin XVIIIA | -2 | 0 | 0 | down |
| ELANE | elastase, neutrophil expressed | -1.88 | 0 | 0 | down |
| SNRPN | small nuclear ribonucleoprotein polypeptide N | -1.73 | 0 | 0 | down |
| CITED4 | Cbp/p300 interacting transactivator with Glu/Asp rich carboxy-terminal domain 4 | -1.72 | 0 | 0 | down |
| MLST8 | MTOR associated protein, LST8 homolog | -1.54 | 0 | 0 | down |
| PINK1-AS | PINK1 antisense RNA | -1.53 | 0 | 0 | down |
| TSPO | translocator protein | -1.44 | 0 | 0 | down |
| COMT | catechol-O-methyltransferase | -1.4 | 0 | 0 | down |
| CST3 | cystatin C | -1.37 | 0 | 0 | down |
| RNPEPL1 | arginyl aminopeptidase like 1 | -1.36 | 0 | 0 | down |
| DOK3 | docking protein 3 | -1.28 | 0 | 0 | down |
| AK2 | adenylate kinase 2 | -1.27 | 0 | 0 | down |
| PTPN18 | protein tyrosine phosphatase, non-receptor type 18 | -1.27 | 0 | 0 | down |
| SPN | sialophorin | -1.26 | 0 | 0 | down |
| TNS3 | tensin 3 | -1.2 | 0 | 0 | down |
| EIF4EBP1 | eukaryotic translation initiation factor 4E binding protein 1 | -1.11 | 0 | 0 | down |
| RNF187 | ring finger protein 187 | -1.1 | 0 | 0.01 | down |
| CFD | complement factor D | -1.09 | 0 | 0 | down |
| MEF2D | myocyte enhancer factor 2D | -1.06 | 0 | 0 | down |
| HPS1 | HPS1, biogenesis of lysosomal organelles complex 3 subunit 1 | -1.06 | 0 | 0.02 | down |
| FES | FES proto-oncogene, tyrosine kinase | -1.01 | 0 | 0.02 | down |
| CHI3L1 | chitinase 3 like 1 | 1.16 | 0.01 | 0.04 | up |
| RRN3 | RRN3 homolog, RNA polymerase I transcription factor | 1.37 | 0 | 0.01 | up |
| RRM2 | ribonucleotide reductase regulatory subunit M2 | 1.78 | 0.01 | 0.07 | up |
